# Effect of Perioperative Dexmedetomidine in Cardiac Surgery: A Narrative Review

**DOI:** 10.7759/cureus.90748

**Published:** 2025-08-22

**Authors:** Zhengmin Ma, Ke Peng, Yufan Yang, Chun Yang, Julie Fowler, Fuhai Ji, Christian Bohringer, Hong Liu

**Affiliations:** 1 Anesthesiology, First Affiliated Hospital of Soochow University, Suzhou, CHN; 2 Anesthesiology, University of California Davis Medical Center, Sacramento, USA

**Keywords:** acute kidney injury, cardiac surgery, dexmedetomidine, myocardial protection, perioperative mortality, postoperative cognitive function, postoperative complications

## Abstract

Cardiac procedures carry a higher perioperative risk than other operations for major adverse cardiovascular events and kidney injury, especially since patients with multiple comorbidities have been accepted as candidates for undergoing surgical treatment. Dexmedetomidine (DEX) is an alpha-2 agonist and has been widely used as an adjuvant anesthetic in clinical anesthesia for many different types of operations, including cardiac surgery. While it can be associated with bradycardia as well as hypotension in hypovolemic patients, DEX has been shown to reduce surgical complications like atrial fibrillation (Afib) and acute kidney injury (AKI), and is associated with an improved survival rate. In this review, we discuss the effect of using perioperative DEX on hemodynamics, arrhythmia, AKI, cognitive function, and surgical outcome in patients undergoing cardiac surgery both with and without cardiopulmonary bypass (CPB), and we review the mechanisms.

## Introduction and background

With the continuous development of new surgical techniques, an increasing number of patients with severe illness who were previously regarded as too high risk for undergoing cardiac surgery are now offered operative intervention. Compared to traditional sternotomy and cardiopulmonary bypass (CPB), a series of less invasive cardiac surgical techniques greatly decreases the physiological stress response of the operation [1, 2]. However, these less invasive procedures are not associated with more stable intraoperative hemodynamics [3]. Compared with other operations, patients undergoing cardiac surgery have a higher incidence of postoperative complications like atrial fibrillation (Afib), acute kidney injury (AKI), and postoperative cognitive dysfunction (POCD), which increase healthcare costs and negatively impact long-term prognosis. [4, 5]. It is therefore important for anesthesiologists to protect the function of vital organs during the operation. Dexmedetomidine (DEX), a highly selective alpha2-adrenergic receptor agonist, was first used for patient sedation in the intensive care unit (ICU) and is now widely used as an adjuvant anesthetic agent during cardiac surgery [6, 7]. As more studies on the protective effect of DEX for vital organs in patients undergoing cardiac surgery are published, the underlying biological molecular mechanisms are gradually being uncovered. This article reviews the current state of knowledge of the protective effect of perioperative DEX in cardiac surgery patients and the potential mechanisms involved.

## Review

Method

In this narrative review, we searched PubMed, OVID Medical Literature Analysis and Retrieval System Online (MEDLINE), and the Excerpta Medica database (Embase) for the relevant literature cited in this review. The time span of the review was from 2006 to 2024. We reviewed a variety of relevant publications, including experimental research, clinical research, and meta-analysis. The following were the relevant keywords used in the literature search: DEX and cardiac surgery, DEX and cardiovascular surgery, DEX and extracorporeal circulation technology, DEX and complications of cardiac surgery, DEX and cardioprotection, and DEX and myocardial injury. The clinical research in this article principally included cohort studies and randomized controlled trials; in addition, important case reports were also cited in this article. No other filters were used. Articles published in languages other than English were excluded.

Dexmedetomidine and myocardial function

DEX and Perioperative Afib

As a selective alpha2-adrenergic receptor agonist, DEX has been shown to decrease the incidence of postoperative ventricular arrhythmia and to prevent postoperative borderline tachycardia in patients undergoing cardiac surgery [8, 9]. New-onset Afib is one of the most common complications after cardiac surgery, with about 30% of patients developing this problem. It has been suggested that DEX, when used as a postoperative sedative, can decrease the risk of developing Afib and result in a lower incidence of postoperative Afib after both on-pump and off-pump cardiac surgery [10-14]. Several meta-analyses also demonstrated that perioperative DEX significantly reduced new-onset Afib [15-18]. In general, patients who received DEX during the perioperative period benefited from an approximately 10% relative risk reduction in the incidence of Afib. This is clinically significant because the onset of new-onset Afib after cardiac surgery is associated with a doubling of the mortality rate.

The potential mechanisms included activation of the dorsal motor nucleus of the vagus nerve and enhanced vagus nerve activity, leading to a decrease in cyclic adenosine monophosphate (cAMP) and the L-type Ca2+ current in myocardial cells, further resulting in prolongation of repolarization and the effective refractory period [19-21]. In addition to enhancing vagus nerve activity, DEX also attenuated sympathetic nervous system activity [22]. DEX inhibited the release of norepinephrine by presynaptic activation of the alpha2-adrenoreceptors in sympathetic nerve endings and subsequent negative feedback on the synaptic vesicles [20]. The effect of DEX on the secretion of hormones such as adrenaline and norepinephrine has been confirmed by multiple studies [23, 24]. In addition, DEX mainly activated the sinoatrial and atrioventricular nodes with the highest density of muscarinic receptors that exert anti-arrhythmic effects [25, 26]. Furthermore, the arrhythmia could occur because of inflammatory action, and DEX indirectly exerts its anti-arrhythmic effect through its anti-inflammatory properties [27, 28]. However, several studies reported that the use of DEX in cardiac surgery did not significantly influence the occurrence of Afib [29-34] and could possibly induce a gradually prolonged PQ interval and complete atrioventricular block [35] (Table 1). The divergent results in these studies may be related to lower doses of DEX and increased amounts of beta agonists given to these patients, as well as differences in the time periods that were chosen for determining the presence of Afib.

**Table 1:** Main clinical studies of dexmedetomidine in perioperative arrhytmia DEX: dexmedetomidine; ICU: intensive care unit; CABG: coronary artery bypass grafting; Afib: atrial fibrillation; POCD: postoperative cognitive dysfunction; RASS: Richmond Agitation Sedation Scale

No.	Author and country	Year	Surgery type	Patient number	Desigen	Treatment	Major findings	Reference
1	Turan A et al., USA	2014	Cardiac surgery	17776; DEX group (n=765), non-DEX group (n=17011)	A retrospective study	DEX was used for patients after surgery in the ICU	DEX used after surgery resulted in fewer atrial arrhythmias	[10]
2	Zi J et al., China	2020	Off-pump CABG surgery	123; DEX group (n=62), propofol group (n=61)	A randomized and double-blinded trial	Patients received continuous sedation of DEX or propofol starting with the initiation of mechanical ventilation	DEX decreased the occurrence of postoperative Afib and inhibited patient anxiety after surgery	[11]
3	Liu X et al., China	2016	Cardiac surgery (patients without prior Afib or atrial flutter)	90; DEX group (n=45), propofol group (n=45)	A randomized controlled trial	After the operation, patients were randomized to receive DEX or propofol, and a targeted sedation score was used during the process of recovery	DEX significantly decreased postoperative Afib	[13]
4	Narisawa A et al., Japan	2015	Cardiovascular surgery	91; DEX group (n=48), non-DEX group (n=43)	A retrospective study	After weaning patients from mechanical ventilation, DEX was used as a sedative agent and was adjusted to a rate of 0.2–0.7μg/kg/h to obtain adequate sedation as assessed by RASS	Adequate nighttime sedation with DEX reduced the incidence of Afib in cardiovascular surgery patients after extubation	[14]
5	Turan A et al., USA	2020	Cardiac surgery	798; DEX group (n=400), placebo group (n=398)	A randomized controlled trial	Patients were given DEX or saline via intravenous pumps from the beginning of the operation until 24h after the operation	DEX use during the whole cardiac surgery process did not influence the incidence of atrial arrhythmias or POCD after the operation	[29]

DEX and Perioperative Hemodynamics

Compared to propofol or fentanyl, the use of DEX alone either before or after intubation led to a higher incidence of hypotension in cardiac patients [36, 37]. However, the combined use of DEX and fentanyl made the hemodynamics more stable compared to when fentanyl was used alone [38]. DEX administration was not associated with an increase in pulmonary artery pressure in pediatric heart surgery patients and noticeably lowered the mean pulmonary arterial pressure (MPAP), the mean arterial pressure (MAP), and the pulmonary capillary wedge pressure (PCWP) throughout the cardiac surgery in patients with pulmonary hypertension [39, 40]. In addition, DEX obviously reduced the impact of surgical stimulation on patient hemodynamics, especially during the bypass time and the post-bypass period [41]. Moreover, DEX could also accelerate the recovery of the microcirculation compared with propofol in patients after valve surgery [42]. However, the use of DEX for complex endovascular aortic aneurysm repair did not result in higher patient satisfaction [43]. The potential protective mechanism of DEX on hemodynamics was demonstrated not only by its bidirectional regulation of coronary artery tone and prevention of maladaptive remodeling but also by its cardioprotective effect under pathological conditions [44, 45].

Mechanisms for the Cardioprotective Effect of DEX

**Figure 1:**
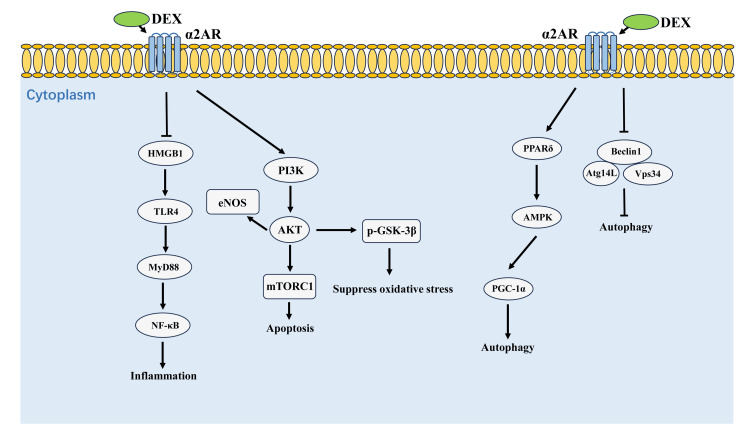
A schematic of proposed mechanisms for the cardioprotection of dexmedetomidine (DEX) α2AR: alpha2 adrenergic receptor; HMGB1: high mobility group box-1; TLR4: toll-like receptor 4; MyD88: myeloid differentiation primary response 88; NF-κB: nuclear factor-kappa B; eNOS: endothelial nitric oxide synthase; PI3K: phosphatidylinositol 3-kinase; AKT: protein kinase B; mTORC1: mechanistic target of rapamycin complex 1; p-GSK-3β: phosphorylation-glycogen synthase kinase-3β; PPARδ: peroxisome proliferator-activated receptors; AMPK: AMP-activated protein kinase; PGC-1α: PHMGB1, high mobility group box 1; TLR4: toll-like receptor 4; PI3K: phoshoinositide 3 kinase; PPARdelta: peroxisome proliferator-activated receptors; GSK3beta: glycogen synthase kinase 3 beta; PGC-1alpha: PPAR-gamma coactivator 1 alpha This figure has been created by the author Zhengmin Ma.

Oxidative stress plays an important role during the myocardial ischemia/reperfusion (I/R) injury process. DEX could alleviate myocardial I/R injury by regulating several different anti-oxidative signaling pathways, for example, the phosphoinositide 3-kinase (PI3K)/protein kinase B (AKT)/glycogen synthase kinase-3β (GSK-3β) axis [46-52]. Many studies also denoted that AKT was involved in DEX myocardial protection through several different mechanisms [53-56]. In addition, DEX also exerted a protective effect by inhibiting reactive oxygen species (ROS)-induced or H₂O₂-induced lesions in the myocardium, which was unrelated to antioxidant enzymes [57, 58]. DEX regulated the autophagy process in cardiomyocytes by either activating the peroxisome proliferator-activated receptors (PPARδ)-AMP-activated protein kinase (AMPK)-PPAR-gamma coactivator 1α (PGC-1α) pathway or by reducing the interactions of the Atg14L-Beclin1-Vps34 complex (Figure 1) [59, 60].

DEX-induced cardioprotection was also mediated by high mobility group box-1 (HMGB1), which was involved in DNA stabilization, gene transcription, and the cholinergic anti-inflammatory pathway [61]. In addition, the use of DEX regulated some microRNA (miRNA) and long noncoding RNA (lncRNA) expression and indeed conferred a protective effect with miR-665, miR-34b-3p, or miR-24 [51, 62-64]. The protective effect of DEX was also regulated through suppressing ferroptosis, pyroptosis, and endoplasmic reticulum (ER) stress [65-68].

DEX and AKI

Postoperative AKI significantly affected patient prognosis. While the etiology of postoperative AKI remains unclear, multiple studies have suggested that timely intervention can be effective in preventing it [69, 70]. Clinical studies have demonstrated that administering DEX prevents AKI following non-cardiac surgery [71-73]. Similarly, DEX was observed to reduce AKI in both adult and pediatric patients undergoing various types of cardiac surgeries [74-83]. These studies indicate that using DEX in the perioperative period in cardiac surgical patients can decrease the incidence of postoperative AKI by 10% to 25% (Table 2). No uniform definition of AKI was used across the studies, but they included Risk, Injury, Failure, Loss (RIFLE), Acute Kidney Injury Network (AKIN), Kidney Disease Improved Global Outcomes (KDIGO), and Society of Thoracic Surgeons (STS) criteria.

**Table 2:** Main clinical studies of dexmedetomidine in AKI DEX: dexmedetomidine; AKI: acute kidney injury; CPB: cardiopulmonary bypass; CABG: coronary artery bypass grafting

No.	Author and country	Year	Surgery type	Patient number	Design	Treatment	Major findings	Reference
1	Cho JS et al., Korea	2016	Cardiac valve surgery	200; DEX group (n=100), control group (n=100)	A prospective, randomized controlled trial	DEX was infused at a rate of 0.4μg/kg/h starting immediately after induction and continued for 24 hours after surgery	DEX during the operation could robustly decrease the severity of postoperative AKI and speed up patient recovery	[74]
2	Zhai MY et al., China	2017	Cardiac valve replacement surgery under CPB	72; DEX group (n=36), placebo group (n=36)	A double-blind, randomized controlled trial	DEX was used from 15 minutes before induction until the end of cardiac surgery; the infusion rate was 0.2μg/kg/h during the operation	DEX could reduce both the incidence of AKI and the severity of renal injury after cardiac surgery	[75]
3	Qiu YQ et al., China	2023	Cardiac valve surgery under CPB	78; DEX group (n=39), control group (n=39)	A randomized controlled trial	Patients in group DEX were given 0.6μg/kg/h intravenously from 10 minutes before induction until 6 hours after surgery	DEX reduced the incidence and severity of postoperative AKI in patients undergoing cardiac valve surgery	[76]
4	Balkanaya OO et al., Turkey	2015	CABG surgery	88; 4μg/cc DEX group (n=31), 8μg/cc DEX group (n=29), placebo group (n=28)	A randomized, triple-blinded, placebo-controlled study	Patients were given DEX or a placebo from the beginning of the operation until 24 hours after the surgery	DEX infusion for sedation after CABG under CPB could prevent AKI	[78]
5	Ji F et al., China	2013	Cardiac surgery	1133; DEX group (n=567), control group (n=566)	A retrospective cohort study	DEX was administered during the post-bypass period in the study group, and the control group was given an equal volume of normal saline	DEX significantly decreased AKI after cardiac surgery, particularly in patients who had normal renal function before the operation	[79]
6	Kwiatkowski DM et al., USA	2016	Congenital heart surgery	204; DEX group (n=102), control group (n=102)	A single-center retrospective matched cohort study	DEX was used on the first postoperative day after surgery	DEX infusion in pediatric patients after congenital heart surgery decreased the incidence of AKI	[80]

It has been suggested that DEX improved renal blood flow, preserved glomerular filtration, increased secretion of water and sodium, and suppressed renin release. DEX administration led to a decrease in the level of renal biomarkers such as blood urea nitrogen (BUN) and serum creatinine (sCr) in an animal study [84]. Similarly, a clinical study suggested that DEX treatment increased intraoperative urine output and decreased the level of BUN and sCr 72 hours post aortic clamping [75]. DEX treatment could also decrease urinary osmolality and maintain hemodynamic stability [85, 86]. In addition, DEX administration inhibited the oxygen consumption rate (OCR) and ROS production in the kidney induced by lipopolysaccharide (LPS) or tumor necrosis factor-alpha (TNF-α) [87, 88]. An in vitro study and an animal study demonstrated that DEX treatment attenuated the toll-like receptor 4 (TLR4) and affected the expression of its downstream inflammatory factor, nuclear factor-kappaB (NF-κB), in renal I/R injury rats [89, 90]. In addition, DEX pretreatment also attenuated renal injury by reducing renal cell apoptosis and downregulating the ER stress [91]. Furthermore, use of DEX exhibited protective mechanisms on AKI via regulating multiple signaling pathways as α2-adrenoreceptor/AMPK/mechanistic target of rapamycin (mTOR), janus kinase (JAK)/signal transducer and activator of transcription (STAT), and GSK‐3β/nuclear factor erythroid 2-related factor 2 (Nrf2) [92-94].

Dexmedetomidine and cognitive function

Cardiac surgery has a marked impact on patients’ cognitive abilities, particularly in elderly patients, leading to POCD, which significantly affects the recovery process. The Mini-Mental State Examination (MMSE), Confusion Assessment Method for the Intensive Care Unit (CAM-ICU), or Montreal Cognitive Assessment (MoCA) was used to assess the cognitive function. Advanced age, preoperative cognitive impairment, and postoperative mechanical ventilation are all significant risk factors for postoperative cognitive impairment. Despite numerous studies exploring potential mechanisms like inflammatory reactions, cerebral hypoperfusion, cerebral microemboli, and anesthetic neurotoxicity, the specific mechanisms involved in cognitive impairment induced by cardiac surgery remain unclear [95, 96]. DEX administration not only decreased the incidence of postoperative delirium but also delayed its occurrence and reduced the duration [97, 98]. Along with its impact on cognitive function, some clinical studies involving DEX infusion in cardiac surgery have denoted that DEX inhibited the perioperative inflammatory response through reducing levels of biomarkers for brain nerve damage or increasing the expression of neuroglobin [99-101]. In addition, several studies demonstrated that DEX could diminish the incidence of postoperative cognitive impairment or delirium compared to midazolam or remifentanil in cardiac surgery with or without CPB [102-105]. However, one study pointed out that the use of DEX during the perioperative period did not significantly affect the delirium of cardiac surgery patients. This could have been due to the relatively low incidence of delirium in the study as a result of the less frequent use of anticholinergic drugs [106]. Collectively, perioperative use of DEX reduced the incidence of POCD in cardiac surgery patients, especially elderly patients, by approximately 20% (Table 3).

**Table 3:** Main clinical studies of dexmedetomidine and cognitive dysfunction DEX: dexmedetomidine; ICU: intensive care unit; POD: postoperative delirium; CPB: cardiopulmonary bypass; POCD: postoperative cognitive dysfunction; Iso: isoflurane; CABG: coronary artery bypass grafting

No.	Author and country	Year	Surgery type	Patient number	Desigen	Treatment	Major findings	Reference
1	Norden JV et al., Germany	2021	Cardiac and major open abdominal surgery	63 (≥60y); DEX group (n=30), placebo group (n=33)	A randomized, double-blind, placebo-controlled trial	DEX was administered during the operation at a rate of 0.2-0.7μg /kg/h, and in the ICU, the infusion rate was adjusted according to the sedation score	DEX use during cardiac surgery significantly decreased the incidence of delirium after the operation	[97]
2	Djaiani G et al., Canada	2016	Cardiac surgery	183 (≥60y); DEX group (n=91), propofol group (n=92)	A single-blinded, prospective, randomized controlled trial	In the ICU, the patients were treated with DEX or propofol infusions. DEX was adjusted to a rate of 0.2-0.7μg/kg/h, and propofol was adjusted to a rate of 25- 50 μg/kg/min	DEX sedation reduced the incidence, delayed the onset, and shortened the duration of POD in elderly patients after cardiac surgery	[98]
3	Zhou MY et al., China	2019	Cardiac valve replacement surgery undergoing CPB	156; DEX group (n=39), ulinastatin group (n=39), DEX+ ulinastatin group (n=39), control group (n=39)	A prospective, randomized, double-blind trial	The dose of ulinastatin was 20,000 UI/kg after induction until the ﬁrst day after surgery; The dose of DEX was 0.4µg/kg/h from induction until 2h prior to extubation	The use of DEX and ulinastatin both reduced the incidence of POCD in patients with cardiac valve surgery	[99]
4	Kang F et al., China	2018	Cardiac valve replacement surgery	97; DEX-Iso group (n=50), Iso group (n=47)	A prospective, randomized, single-blind study	DEX was given as a 0.6μg/kg bolus over 15 minutes and continued at 0.2μg/kg/h until the end of surgery	The use of DEX decreased the biochemical markers of brain injury after cardiac surgery	[100]
5	Park JB et al., Korea	2014	Cardiac surgery under CPB	142; DEX group (n=67), remifentanil group (n=75)	A prospective randomized controlled study	After surgery, the patients were treated with DEX (loading dose, 0.5μg/kg; maintenance dose, 0.2 to 0.8μg/kg/h) or remifentanil (range, 1,000 to 2,500μg/h)	DEX was associated with a lower rate of delirium after cardiac surgery when used as a postoperative sedative agent	[102]
6	Likhvantsev VV et al., Russia	2021	Cardiac surgery (CABG surgery, valve surgery, or combined surgery) with CPB	175; DEX group (n=87), placebo group (n=88)	A randomized controlled trial	Infusion of DEX was started at induction and continued until the beginning of weaning of ventilation in the ICU. Infusion rates were 0.4 to 1.4μg/kg/h	DEX administered during and after general anesthesia for cardiac surgery with CPB reduced the rate of postoperative delirium and ICU length of stay	[105]

DEX inhibited neuronal excitation, alleviated the electrophysiological dysfunction of neuronal mitochondrial membranes, and promoted the recovery of neurogenesis [107-109]. Other studies suggested that DEX could alleviate postoperative cognitive dysfunction via activating the cholinergic pathway and the locus coeruleus norepinephrine system [110, 111]. Furthermore, DEX administration regulated microglial activation and inhibited neuroinflammation by acting on multiple miRNAs, lncRNAs, or circular RNA [112-118]. Use of DEX effectively inhibited the expression level of several inflammatory factors like interleukin-6 (IL-6), IL-1beta (IL-1β), and TNF-α in the brain during the perioperative period [119, 120]. In addition, several studies suggested that DEX could influence neuronal nitric oxide synthase (nNOS), the NOD-like receptors (NLR) family, the pyrin domain containing 3 (NLRP3) inflammasome, or the HMGB1/receptor of advanced glycation end-products (RAGE)/NF-κB signaling pathway to alleviate postoperative inflammatory reaction [121-125]. Moreover, DEX played a neuroprotective role during different surgeries by alleviating the neurotoxicity of various intravenous or inhalation anesthetics, including sevoflurane, isoflurane, propofol, and ketamine [126-134]. Noteworthy, recent research implied that the effect of DEX on cognitive impairment after cardiac surgery could result from the interaction of multiple mechanisms [135].

Dexmedetomidine and postoperative mortality

Perioperative DEX has been shown to improve the five-year survival rate after cardiac surgery between approximately 13% and 20% [136]. Other studies also demonstrated that the use of DEX improved the short and medium-term survival rates after cardiac or noncardiac surgery (30 days, one year, or two years) [137-140]. In the retrospective studies, all data were adjusted with propensity scores, inverse probability of treatment weighting (IPTW), or overlap weighting approaches. DEX improved the postoperative survival rate by minimizing heart, kidney, and brain injuries after cardiac surgery (Table 4) [137].

**Table 4:** Main clinical studies of DEX and postoperative mortality DEX: dexmedetomidine; CABG: coronary artery bypass graft; ICU: intensive care unit; CPB: cardiopulmonary bypass

No.	Author and country	Year	Surgery type	Patient number	Desigen	Treatment	Major findings	Reference
1	Peng K et al., China	2021	Cardiac surgery	2068; DEX group (n=1029), non-DEX group (n=1039)	A retrospective cohort study	DEX was used before or immediately after CPB and lasted for less than 24 hours	The use of DEX improved the five-year survival rate of patients undergoing cardiac surgery	[136]
2	Ji F et al., China	2013	Cardiac surgery	1134; DEX group (n=568), non-DEX group (n=566)	A retrospective study	Cardiac surgical patients did or did not receive DEX during the surgery	The use of DEX improved one-year postoperative survival in patients undergoing cardiac surgery and reduced the incidence of various postoperative complications	[137]
3	Ji F et al., China	2014	CABG surgery	724; DEX group (n=345), non-DEX group (n=379)	A retrospective study	DEX was used immediately after CPB and continued for less than 24 hours after surgery	The use of DEX during CABG surgery improved in-hospital, 30-day, and one-year survival rates	[138]
4	Zhang DF et al., China	2019	Noncardiac surgery	700; DEX group (n=350), placebo group (n=350)	A randomized controlled trial	DEX was used within one hour after ICU admission until the first day after surgery	In elderly patients undergoing non-cardiac surgery, low-dose DEX increased survival up to two years	[140]

Postoperative survival rates are influenced by multiple interrelated factors. While current research has predominantly examined all-cause mortality, it should be noted that any single factor is unlikely to independently produce statistically significant alterations in overall survival outcomes. The incidence of DEX-associated adverse effects, including hypotension and severe bradycardia, remains relatively low. Consequently, current evidence is insufficient to establish a significant correlation between DEX-related side effects and postoperative survival outcomes.

Study limitations

Current evidence regarding DEX utilization in high-risk patients undergoing minimally invasive cardiac procedures (e.g., transcatheter aortic valve replacement or mitral valve clipping) remains limited. Particularly scarce are multi-center prospective studies with an adequate sample size to ensure statistical power. There was significant heterogeneity in study design, patient populations, dosing protocols, and measured endpoints across the reviewed studies. This represents a critical knowledge gap that warrants further investigation through rigorously designed clinical trials.

## Conclusions

DEX used as an anesthesia adjuvant has been shown to maintain intraoperative hemodynamic stability and improve postoperative recovery and long-term outcomes. Inhibition of myocardial oxygen consumption and attenuation of sympathetic nervous system activity play a fundamental role in its myocardial protection during cardiac surgery. Furthermore, the anti-inflammatory properties of DEX provide organ protection and reduce the incidence of postoperative AKI and POCD. While DEX may be associated with bradycardia as well as hypotension, especially in hypovolemic patients, more extensive perioperative use of DEX in cardiac surgery has the potential to significantly improve outcomes in this high-risk patient population. 
